# Sudan virus disease outbreak in Uganda: urgent research gaps

**DOI:** 10.1136/bmjgh-2022-010982

**Published:** 2022-12-30

**Authors:** Susan Khader Ibrahim, Duduzile Edith Ndwandwe, Katherina Thomas, Louise Sigfrid, Alice Norton

**Affiliations:** 1 GloPID-R Research and Policy Team, Pandemic Sciences Institute, University of Oxford, Oxford, UK; 2 Cochrane South Africa, South African Medical Research Council, Cape Town, South Africa; 3 Walimu, NGO, Kampala, Uganda; 4 ISARIC Global Support Centre, Pandemic Sciences Institute, University of Oxford, Oxford, UK

**Keywords:** Public Health, Infections, diseases, disorders, injuries, Viral haemorrhagic fevers, Review

## Abstract

The Sudan ebolavirus (SUDV) outbreak highlights our ongoing vulnerability to re-emerging high-consequence infectious diseases. Although the Minister of health in Uganda has initiated public health measures in collaboration with neighbouring countries and with support of the WHO, cases have continued to spread to several regions including the capital. The ongoing transmission, uncertain case numbers and no licensed vaccine or therapeutics available are a cause for concern. We searched four databases for SUDV research using the search terms “SUDV”, “Sudan Virus” and “Ebola Sudan”. Our analysis identified only 20 SUDV research studies. Most were implemented in the USA and only one in Uganda. Nine studies were on therapeutics, eight on vaccines, one on diagnostics, one in one health and one in social science. Our data highlight a lack of SUDV research and an urgent need for investment to identify an effective vaccine, and optimal supportive care and therapeutic strategies for all at risk groups as a key research priority. Research investments should be prioritised into vaccines and treatment strategies that will be accessible to high-risk populations in affected regions during the outbreak, to protect populations, improve individual outcomes and facilitate outbreak control.

**Summary box**

- Uganda is facing an Ebola outbreak caused by the Sudan ebolavirus (SUDV) in a health system currently responding to multiple emergencies.
- The Sudan virus disease (SVD) mortality rate in previous outbreaks ranged from 41% to 100%.
- Uganda has faced multiple Ebola outbreaks in the past, but a lack of vaccines and therapeutics against SVD plus cases in several regions, including the capital, is a cause for concern.
- We provide an overview of up-to-date information on the SVD outbreak in Uganda and present the results from a rapid review of existing research on SVD and detail the existing vaccine candidates that have reached the point of human trials and ongoing research studies to inform research gaps.
- Our data highlight a need for urgent investments into research on SUDV to identify effective vaccine and optimal treatment strategies and community engagement to support trial implementation.
- The data also emphasise our vulnerability to infectious disease outbreaks and the need to strengthen our health systems and research response capacity and capability globally to manage concurrent emergencies, with a focus on resource-deprived settings.

## Background

On 20 September 2022, Uganda declared the first confirmed case of Sudan virus disease (SVD) in the current outbreak.^1 2^ By 12 October, there were 54 confirmed cases^3^ with 19 deaths, 4 of which were in healthcare workers.^4 5^ Ebola is a severe disease, with high mortality risk, first identified in 1976 when two simultaneous outbreaks occurred in South Sudan and the Democratic Republic of Congo (DRC).^6^ Ebolaviruses belong to the viral family Filoviridae.^1 7 8^ There are six known ebolaviruses, four of which can cause human disease (Bundibugyo, Sudan, Taï Forest and Zaire viruses).^7 8^ Ebola outbreaks have most commonly been caused by the Zaire ebolavirus and Sudan ebolavirus (SUDV).^9 10^ Since May 2019, Ebola caused by the SUDV is known as SVD.^1 2^ Sudan and Uganda have predominantly been affected by SUDV outbreaks.^10^ Uganda has had six previous ebolavirus disease outbreaks, four of which were SUDV outbreaks in 2000 and 2011 and two in 2012.^1 9 10^ The most severe of those was the 2000 outbreak with 425 recorded cases and 224 deaths.^9 10^ The aims of this article are to provide an overview of the SVD outbreak and response in Uganda and to present findings from our rapid analysis of existing research to identify research gaps to inform research response prioritisation.

## Evolution of the SUDV outbreak in Uganda

Uganda declared an outbreak of SVD on 20 September 2022 with its first confirmed case in a 24-year-old man in the Mubende district.^1 2 11^ The case presented to two healthcare facilities between 11 and 15 September before being referred to the referral regional hospital where he was diagnosed with SUDV on 19 September.^1 2^ It is estimated that SUDV has been circulating in the Mubende district for 3 weeks prior to this first case being detected, with reports of unexplained community deaths during the first 2 weeks of September and traditional burials for those who died.^1 2^

**Figure 1:**
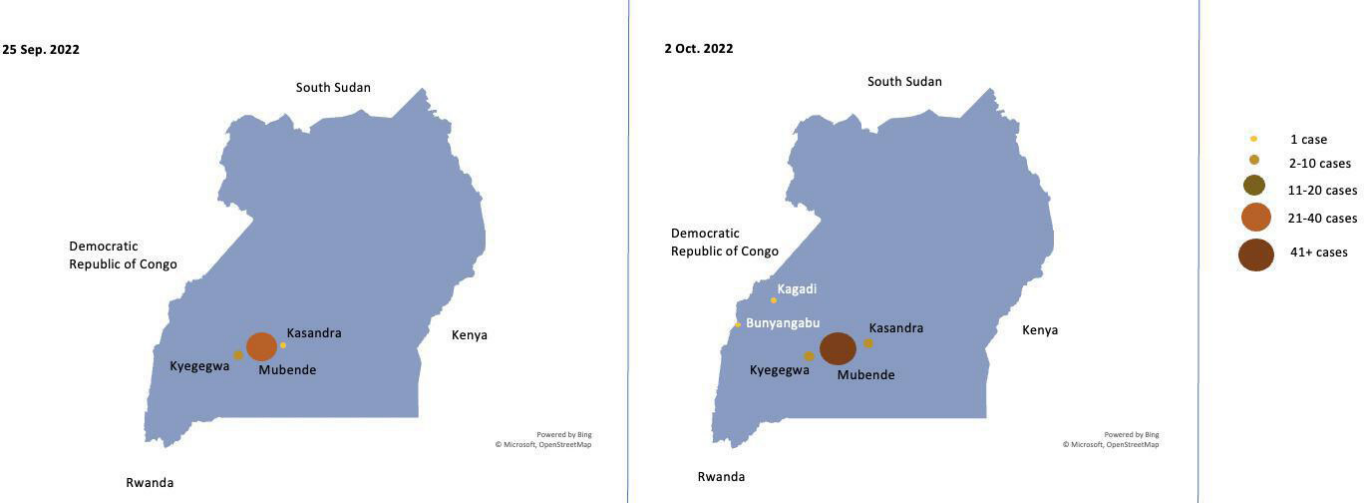
Geographical transmission of SVD cases in Uganda (25 September–2 October 2022). The map shows the total number of confirmed and probable cases of SVD reported from Uganda by district. Data source: WHO Regional Office for Africa Weekly Bulletin on Outbreaks and Other Emergencies,^2 12^ created in Microsoft Excel. SVD, Sudan virus disease.

By 25 September, 36 cases were reported (18 confirmed and 18 probable) from three districts with 35 of these admitted to the hospital.^2^ The median age of these cases were 26 years old (range 1–63 years old). By 2 October, the case reports had increased to 43 confirmed cases from five districts (figure 1).^12^ The Mubende district remains the most heavily affected with 36 confirmed cases.^12^ By 10 October, the Uganda Ministry of Health reported 48 confirmed cases and a death from SUDV in its capital, Kampala, by an individual who did not isolate after a relative died in an affected district, but instead visited a traditional healer.^4 13 14^ By 12 October, there were 54 confirmed cases and 19 deaths reported.^3^ The rise and geographical spread of the cases are a cause of concern.^9^

This outbreak initially predominantly affected gold miners, who are a highly mobile population, which may increase the risk of spread to other districts and bordering countries.^2^ One of the affected districts (Bunyangabu) borders the DRC.^12 15^ The transmission into several districts highlights a need to strengthen preparedness and readiness activities across the country and in collaboration with neighbouring countries.^12^

## Transmission, clinical presentation and case fatality

Ebolaviruses are primarily transmitted to humans through close contact with blood, secretions, organs or other bodily fluids of infected humans or animals and contaminated surfaces and materials.^16^ The incubation period ranges from 2 days to 21 days.^16^ An increase in population size, the rise of urbanisation and the interconnectedness of travel can expand the spread of filovirus disease beyond endemic regions.^17^

The symptoms of Sudan and Zaire ebolavirus are similar.^1^ Patients generally present with fever, fatigue, muscle pain, headache and sore throat, followed by vomiting, diarrhoea, rash, and/or symptoms of impaired kidney and liver function.^16 18^ More severe illness can include internal and external bleeding, multiorgan failure, encephalopathy, respiratory distress, shock and spontaneous abortion in pregnant women.^16^ Diagnosing SVD can be challenging as the initial symptoms resemble other common infectious diseases including malaria.^6^ Diagnosis can be made using reverse transcriptase–PCR.^1 6 19^ More novel laboratory tests also exist, such as OraQuick Ebola rapid antigen test approved by the US Food and Drug Administration.^19^ This test detects antigens from viruses in the *Ebolavirus* genus but cannot differentiate between viruses^19^ and is deemed not sensitive enough to detect SUDV.^20^

The average Ebola case fatality rate is estimated around 50% with rates varying from 25% to 90% in past outbreaks.^6^ The case fatality for SVD ranged from 41% to 100% in past outbreaks.^1^ Early data from the ongoing outbreak of SVD in Uganda showed a case fatality rate of 46% based on 63 confirmed and probable cases, with 29 deaths and 4 recoveries at the time of the estimation.^12^ Healthcare workers are at high risk of exposure; by 5 October, there were four reported healthcare worker deaths from SUDV in Uganda.^5^

Currently, there are no licensed vaccines or therapeutics for SUDV.^1^ There are monoclonal antibodies (mAB114 and REGN-EB3, also known as Ansuvimab or Ebanga and Inmazeb, respectively) licensed for treatment of Ebola caused by the Zaire ebolavirus but not for SUDV.^21^ Merck’s (MSD) rVSV-ZEBOV (ERVEBO) vaccine is ready to be deployed for outbreaks of Zaire ebolavirus; however, Zaire ebolavirus is genetically distinct from Sudan virus, and available evidence suggests that it will not provide cross protection against SUDV.^1 11 18^ The Johnson & Johnson’s Zabdeno/Mvabea vaccine is a two-dose vaccine, with the first dose priming and the second dose boosting the immune response against the Zaire ebolavirus.^18^ Additional antigens, including Sudan virus, are present in the second dose and may provide protection based on available evidence from a non-human primate model; however, it has not been tested against SUDV in humans, and due to the second dose being administered after 56 days, it is less ideal for outbreak response.^1 18^

With a lack of vaccines and therapeutics, access to optimal supportive care guided by physiological monitoring and treatment of secondary complications are key for improving survival rates.^1^ The prolonged Ebola outbreak caused by Zaire ebolavirus from 2013 to 2016 in West Africa allowed for the evaluation of supportive care.^22^ This included evaluating care such as volume resuscitation, symptom control, monitoring of glucose, electrolyte levels and organ dysfunction, and treatment of coinfections.^22^ This data informed the WHO 2019 guideline on optimised supportive care for Ebola.^22^ In August 2022, the WHO released a linked guideline specifically focused on therapeutic treatment recommendations for the Zaire ebolavirus.^23^ It is not yet known if these treatments are effective against SUDV.

## SUDV research

Limited research on SUDV exists partly because outbreaks have been sporadic.^9^ The GloPID-R research and policy team conducted a rapid search of the World RePORT, European Union Clinical Trials Registry, Pan African Clinical Trial Registry (PACTR) and WHO International Clinical Trials Registry Platform databases using search terms “SUDV”, “Sudan virus” and “Ebola Sudan”.^24–26^ No time restrictions were applied to the search to rapidly identify any existing research related to SUDV. Our searches identified 20 studies as of 15 November 2022; 9 were focused on therapeutics, 8 on vaccines, 1 on social sciences, 1 on diagnostics and 1 on one’s health. There were no trials registered in PACTR as of 20 October 2022 (online supplemental appendix 1).

10.1136/bmjgh-2022-010982.supp1 Supplementary data

Of the nine studies that related to therapeutics, eight were focused on monoclonal antibodies and one at ebolavirus cell entry inhibitors. Only two studies focused on therapeutic development specific to SUDV; the remaining seven aimed to develop therapeutics that covered a broad spectrum of ebolaviruses. Eight studies were based in the USA; the other was a collaborative study with seven countries involved, including Uganda.

Of the eight studies that related to vaccines, two had conducted human studies. One of these, led by the University of Oxford, was a phase I and Ib study which began enrolment of participants in the UK in November 2021 and Tanzania in March 2022. It aimed to determine the safety and immunogenicity of the bivalent CHAdOx1 vectored vaccine. The other was a completed phase I clinical trial evaluating the safety, tolerability and immunogenicity of two doses of an Ebola Sudan chimpanzee adenovirus vector vaccine led by the Sabin Vaccine Institute. The remaining six studies identified were all focused on supporting the development of broad-spectrum vaccines. Four of these studies were based in the USA; one was a collaboration between Canada and the USA; and one was a collaboration between the UK and the USA (online supplemental appendix 1).

The WHO R&D blueprint team have been tracking progress of SUDV vaccine research. So far, they have registered 24 SUDV vaccine candidates (as of 28 September 2022).^27^ These are indicated at different stages of development (clinical (n=1), phase I (n=2) and preclinical (n=21)).^27^ Four vaccine candidates have progressed to the point where human trials have begun or can begin soon, including the two described earlier (table 1).^28–40^ The WHO has stated that three of these (cAd3, cAdOX1 and rVSV SUDV GP) are under consideration for clinical trials in Uganda.^37^ The cAd3 vaccine is a single-dose vaccine developed partly by the US National Institute of Allergy and Infectious Diseases and now licensed to the Sabin Vaccine Institute.^9 27–29 32 33^ It has shown protection against SUDV in non-human primates.^9 29^ One hundred doses of this vaccine are being shipped to Uganda with advice to prioritise vaccination for healthcare workers.^9 33^ The cADOX1 vaccine is another single-dose vaccine developed by the Jenner Institute at the University of Oxford.^32^ The rVSV SUDV GP vaccine developed by MSD is a single-dose vaccine specific to SUDV, using a replication-competent, live, attenuated recombinant vesicular stomatitis virus (rVSV) vaccine construct similar to that used for ERVEBO (Ebola Zaire Vaccine, Live). The rVSV SUDV vaccine has shown efficacy in non-human primates.^38 39 41^ MSD aims to deliver 50 000 doses to Uganda by the end of 2022 via the International AIDS Vaccine Initiative, a non-profit scientific research organisation, for phase I and efficacy studies.^38 39^

**Table 1:** SUDV vaccine candidates that have or are rapidly progressing to human trials as of 15 November 2022

Vaccine name	Studies conducted	Lead institution
cAd3-EBO S (VRC-EBOADC086-00-VP	Phase I clinical trials in Uganda and USA, completed Planning phase II trial	Sabin Vaccine Institute (NIAID)
ChAdOx1 biEBOV	Phase I clinical trials in the UK and Tanzania (currently recruiting)	University of Oxford
rVSV SUDV GP	Preclinical phase, rapidly preparing for phase I trial and efficacy studies	MSD
Ad26.ZEBOV, MVA-BN-Filo	Phase III clinical trial in Sierra Leone, completed Vaccine approved by the European Medicines Agency (EMA)	Johnson & Johnson

*The Johnson & Johnson vaccine is a two-dose regimen with the second dose providing protection against SUDV. The second dose is administered after 56 days of the first dose.^1^

EMA, European Medicines Agency; MSD, Merck; NIAID, National Institute of Allergy and Infectious Diseases; SUDV, Sudan ebolavirus.

These data highlight an urgent need for research investments into trials to identify vaccines and therapeutics for SVD, as well as optimal supportive care strategies. It also highlights the need for broader research studies relating to the wider understanding of SVD, including epidemiology and social science aspects. Access to effective vaccines, together with stringent public health and hospital infection control methods, is key for outbreak control, including therapeutics and supportive care for improving survival rates.

### National public health response

**Figure 2:**
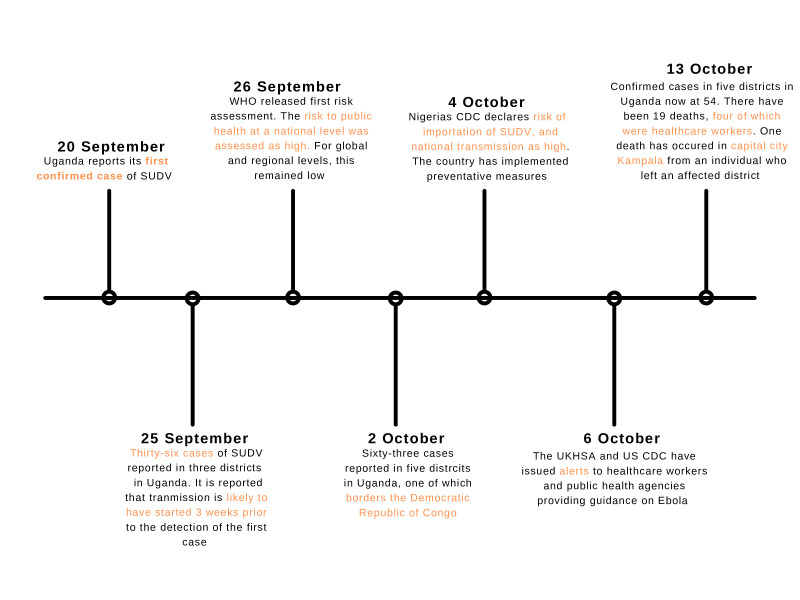
An overview of the early public health response. CDC, Centers for Disease Control and Prevention; SUDV, Sudan ebolavirus; UKHSA, UK Health Security Agency.

Multiple task forces including the Uganda MOH and WHO have been holding regular meetings since the 2022 SUDV outbreak was declared and have visited the affected districts to evaluate and orientate response activities.^12^ Epidemiological investigations in the affected districts identified 884 contacts with 60% of these followed up as of 2 October.^12^ Mobile laboratory clinics set up at Mubende Regional Referral Hospital are processing samples.^9 12^ Uganda are training staff in SUDV reporting, contact tracing and infection, prevention, control procedures and safe burials, and have initiated community awareness campaigns via media (figure 2).^12^

### WHO risk assessment

The WHO has assessed the public health risk of SUDV in Uganda as very high at the national level, high at the regional level and low at the global level as of 10 November 202 2.^42^ This is based on the following^42^:

- Lack of an authorised vaccine for SUDV.
- SUDV has affected a highly mobile population group, and cases have spread to multiple districts with high populations.
- Health clinics where patients presented had limited infection prevention and control measures.
- The possibility for missed contact cases.
- Community engagement in affected districts reported as challenging.
- Uganda has local capacity and resources for an effective response to SUDV; however, the country is dealing with multiple emergencies, including COVID-19, anthrax, yellow fever, food insecurity and flooding. If cases continue to rise and spread to other districts, the system could be overwhelmed.

### Regional and international alerts

No cases have been identified in other countries to date. However, internationally, some countries have issued advice to public health providers and healthcare workers to be alert to risk of travel imported cases, with guidance on identification and diagnostics, and advising on travel history in patients presenting with Ebola/SUDV symptoms.^43 44^ Based on the international risk assessment, the risk to other countries is low, and there are currently no recommendations on travel restrictions by WHO. Many countries have initiated travel-related guidance. The USA has implemented screening of passengers arriving from Uganda.

The Nigeria Centre for Disease Control (NCDC) declared a high risk of importation of SUDV and impact on national health on 4 October.^45^ The high risk of importation assessment was based on high travel volume between Nigeria and Uganda, and the risk of onward transmission nationally due to upcoming large gatherings and religious festivals.^45^ The NCDC has put preventative measures in place, including development of an incident action plan, risk communication and engagement, trained rapid response teams put on standby and follow-up of passengers from Uganda up to 21 days after their arrival in Nigeria.^45^ The NCDC has advised avoiding non-essential travel to Uganda.^46^

The government of Uganda, the Africa Centres for Disease Control and Prevention and WHO hosted a high-level emergency meeting on cross-border collaboration for preparedness and response to Ebola disease outbreaks in Kampala on 12 October.^3^ Ministers of Health and government representatives from nine African countries agreed on joint measures to stop the potential spread of the ongoing outbreak in Uganda and beyond its borders.^3^ These include disease surveillance, contact tracing and monitoring, prompt alert notification, information sharing, joint trainings of emergency responders and simulation exercises to enhance preparedness and response.^3^

## Conclusion

The re-emergence of SUDV in Uganda with rapid transmission into multiple districts with high mortality rate re-emphasises our global vulnerability to re-emerging infections and reinforces the need for investments to strengthen our preparedness to combat high-consequence infectious diseases. Without effective treatments, supportive care guided by evidence and physiological monitoring is key for improving survival rates.

The regional response to SUDV has been swift in implementation of public health control; however, despite previous outbreaks, our data highlight a lack of prior and active SUDV research, and a need for urgent investments to identify effective vaccines and therapeutics to improve outcomes. There are certain research activities which can only be undertaken while a virus is circulating in the human population, such as therapeutic and vaccine trials, evaluation of public health measures and community perceptions, and understanding transmission dynamics and epidemiology. Optimising surveillance and evaluation of point of care diagnostics and research across these areas now need to be rapidly activated.^47 48^

Our data highlight our global vulnerability to re-emerging infectious disease outbreaks and the need to improve our preparedness for high-consequence infectious diseases through investments into global research capacity, medical countermeasure platforms and strategic clinical research networks with capacity to respond at the outset of outbreaks, with a focus on deprived resource settings, to protect populations, health systems and improve epidemic outcomes.

## Data Availability

All data relevant to the study are included in the article or uploaded as supplementary information.

## References

[1] World Health Organization. Ebola Disease caused by Sudan virus - Uganda. Disease Outbreak News, 2022. Available: https://www.who.int/emergencies/disease-outbreak-news/item/2022-DON410 [Accessed 11 Oct 2022].

[2] World Health Organization Regional Office for Africa. Weekly Bulletin on outbreaks and other emergencies. week 39: 19-25 September 2022. Available: https://extranet.who.int/iris/restricted/bitstream/handle/10665/363303/OEW39-1925092022.pdf [Accessed 11 Oct 2022].

[3] World Health Organization. African health ministers take steps to curb Ebola disease outbreak. Uganda: World Health Organization, 2022. https://www.afro.who.int/countries/uganda/news/african-health-ministers-take-steps-curb-ebola-disease-outbreak

[4] European Centre for Disease Prevention and Control. Ebola outbreak in Uganda under ECDC monitoring. ECDC Media Centre, 2022. https://www.ecdc.europa.eu/en/news-events/ebola-outbreak-uganda-under-ecdc-monitoring

[5] Isaac D. Fourth Ugandan health worker dies from Ebola. ripples Nigeria, 2022 Oct 5. Available: https://www.ripplesnigeria.com/fourth-ugandan-health-worker-dies-from-ebola/ [Accessed 11 Oct 2022].

[6] World Health Organization. Ebola virus disease. Newsroom, 2021. https://www.who.int/en/news-room/fact-sheets/detail/ebola-virus-disease

[7] Centers for Disease Control and Prevention. What is Ebola virus disease? Ebola (Ebola Virus Disease), 2021. https://www.cdc.gov/vhf/ebola/about.html

[8] European Centre for Disease Prevention and Control. Factsheet about Ebola virus disease. ECDC: Ebola virus disease Facts, 2022. https://www.ecdc.europa.eu/en/infectious-disease-topics/z-disease-list/ebola-virus-disease/facts/factsheet-about-ebola-virus

[9] Kozlov M. Ebola outbreak in Uganda: how worried are researchers? Nature 2022;597. doi:10.1038/d41586-022-03192-8. [Epub ahead of print: 07 Oct 2022] https://www.nature.com/articles/d41586-022-03192-8 36207520

[10] Centers for Disease Control and Prevention. Hisotry of Ebola outbreaks. Ebola (Ebola Virus Disease), 2022. https://www.cdc.gov/vhf/ebola/history/chronology.html?CDC_AA_refVal=https%3A%2F%2Fwww.cdc.gov%2Fvhf%2Febola%2Foutbreaks%2Fhistory%2Fchronology.html

[11] Travel Health Pro. Ebola: new outbreak in Uganda. Latest News, 2022. https://travelhealthpro.org.uk/news/661/ebola-new-outbreak-in-uganda

[12] World Health Organization Regional Office for Africa. Weekly Bulletin on outbreaks and other emergencies, 2022. Available: https://apps.who.int/iris/bitstream/handle/10665/363400/OEW40-260902102022.pdf [Accessed 11 Oct 2022].

[13] Ministry of Health. Ebola. Republic of Uganda Ministry of Health, 2022. https://www.health.go.ug/ebola/

[14] Uganda leader cracks down on traditional healers to stem Ebola. France 24, 2022 Oct 13. Available: https://www.france24.com/en/live-news/20221013-uganda-leader-cracks-down-on-traditional-healers-to-stem-ebola [Accessed 13 Oct 2022].

[15] Centers for Disease Control and Prevention. September 2022 Uganda, Mubende district. Ebola (Ebola Virus Disease), 2022. https://www.cdc.gov/vhf/ebola/outbreaks/uganda/2022-sep.html

[16] ICD-11 for Mortality and Morbidity Statistics. 1D60.0 Ebola disease. Certain zoonotic viral diseases in ICD-11 Coding Tool, 2022. https://icd.who.int/ct11/icd11_mms/en/release

[17] Emperador DM, Mazzola LT, Wonderly Trainor B, et al. Diagnostics for filovirus detection: impact of recent outbreaks on the diagnostic landscape. BMJ Glob Health 2019;4:e001112. 10.1136/bmjgh-2018-001112 PMC6407532 30899573

[18] Geddes L. What do we know about the outbreak of Ebola in Uganda so far? Gavi, 2022. https://www.gavi.org/vaccineswork/what-we-know-about-outbreak-ebola-uganda-so-far

[19] Broadhurst MJ, Brooks TJG, Pollock NR. Diagnosis of Ebola virus disease: past, present, and future. Clin Microbiol Rev 2016;29:773–93. 10.1128/CMR.00003-16 27413095 PMC5010747

[20] eBioMedicine. Ebola outbreak in Uganda: urgent call for better prevention and surveillance. EBioMedicine 2022;85:104366. 10.1016/j.ebiom.2022.104366 36371089 PMC9669767

[21] Heilprin J. Who recommends two monoclonal antibodies for Ebola treatment; calls to expand access in developing countries. Health Policy Watch Independent Global Health Reporting, 2022. https://healthpolicy-watch.news/whos-first-ebola-treatment-guidelines-recommend-two-monoclonal-antibodies/

[22] WHO Emergency Programme Clinical Management Team. Optimized supportive care for Ebola virus disease clinical management standard operating procedures, 2019. Available: https://apps.who.int/iris/handle/10665/325000

[23] World Health Organization. Therapeutics for Ebola virus disease. Geneva: World Health Organization, 2022. https://www.who.int/publications/i/item/9789240055742

[24] World Health Organization. International clinical trials registry platform (ICTRP), 2022. Available: https://www.who.int/clinical-trials-registry-platform [Accessed 12 Oct 2022].

[25] World report. Available: https://worldreport.nih.gov/wrapp/#/search?searchId=63469aec044d0c13965be5c0 [Accessed 12 Oct 2022].

[26] European Union. Eu clinical trials register. EU Clinical Trials, 2022. https://www.clinicaltrialsregister.eu/ctr-search/search

[27] World Health Organization. Sudan Virus Vaccine Tracker - List of vaccine candidates in research & development. Publications, 2022. https://www.who.int/publications/m/item/sudan-virus-vaccine-tracker-list-of-vaccine-candidates-in-research-development

[28] ClinicalTrials.gov.. Ebola Sudan chimpanzee adenovirus vector vaccine in healthy adults. ClinicalTrials.gov, 2021. https://clinicaltrials.gov/ct2/show/NCT04041570

[29] Ledgerwood JE, DeZure AD, Stanley DA, et al. Chimpanzee adenovirus vector Ebola vaccine. N Engl J Med 2017;376:928–38. 10.1056/NEJMoa1410863 25426834

[30] The Jenner Institute. Eboal virus vaccine study (EBL07. Recruiting Trials, 2022. https://www.jenner.ac.uk/volunteer/recruiting-trials/ebola-virus-vaccine-study-ebl07

[31] ClinicalTrials.gov. A study of a new vaccine against two types of Ebola. Study Record Detail, 2021. https://clinicaltrials.gov/ct2/show/NCT05079750

[32] Sabin Vaccine Institute. Vaccine Research & Development. Our Programs, 2022. https://www.sabin.org/our-impact/programs/vaccine-research-and-development/

[33] Branswell H. Ebola experimental vaccine trial may begin soon in Uganda. STAT, 2022. https://www.statnews.com/2022/09/29/ebola-experimental-vaccine-trial-may-begin-soon-in-uganda/

[34] Johnson & Johnson. Johnson & Johnson Announces European Commission Approval for Janssen’s Preventive Ebola Vaccine. Johnson & Johnson. Johnson & Johnson, 2020. https://www.janssen.com/emea/sites/www_janssen_com_emea/files/jnj_ebola_ec_approval_v19.0_imr_approval_010720.pdf

[35] Johnson & Johnson. Johnson & Johnson Ebola Vaccine Regimen Demonstrated Robust and Durable Immune Response in Adults and Children in Data Published in The Lancet Infectious Diseases. Innovation, 2021. https://www.jnj.com/johnson-johnson-ebola-vaccine-regimen-demonstrated-robust-and-durable-immune-response-in-adults-and-children-in-data-published-in-the-lancet-infectious-diseases

[36] Tiemessen MM, Solforosi L, Dekking L, et al. Protection against Marburg virus and Sudan virus in NHP by an Adenovector-Based trivalent vaccine regimen is correlated to humoral immune response levels. Vaccines 2022;10:1263. 10.3390/vaccines10081263 36016151 PMC9412258

[37] The World Health Organization. Ebola disease caused by Sudan ebolavirus - Uganda [Internet]. Disease Outbreak News, 2022. Available: https://www.who.int/emergencies/disease-outbreak-news/item/2022-DON423

[38] Cohen J. Merck locates frozen batch of undisclosed Ebola vaccine, will donate for testing in Uganda’s outbreak. Science, 2022. https://www.science.org/content/article/uganda-may-use-destroyed-ebola-vaccine-merck-fight-its-growing-outbreak

[39] IAVI. IAVI to accelerate promising investigational Sudan ebolavirus vaccine development for potential research and response. Press Releases, 2022. https://www.iavi.org/news-resources/press-releases/2022/iavi-to-accelerate-promising-investigational-sudan-ebolavirus-vaccine-development-for-potential-outbreak-research-and-response

[40] Precision vaccinations staff. Ebola vaccines 2022. Precision vaccinations. Ebola Vaccines, 2022. https://www.precisionvaccinations.com/ebola-vaccines

[41] MERCK. Merck Responds to Sudan Ebolavirus Outbreak in Uganda with Plans to Produce and Donate Investigational Vaccine Doses for IAVI’s Vaccine Development Program. Company statement., 2022. Available: https://www.merck.com/news/merck-responds-to-sudan-ebolavirus-outbreak-in-uganda-with-plans-to-produce-and-donate-investigational-vaccine-doses-for-iavis-vaccine-development-program/

[42] The World Health Organization. Ebola disease caused by Sudan virus - Uganda. Disease Outbreak News, 2022. Available: https://www.who.int/emergencies/disease-outbreak-news/item/2022-DON421

[43] Waite T, Hopkins S, Powis S. Public health message to all NHS service providers regarding Ebola virus outbreak in Uganda (Sudan ebolavirus. UK Health Security Agency, 2022. https://www.gov.uk/government/publications/ebola-outbreak-of-sudan-ebolavirus-in-uganda/public-health-message-to-all-nhs-service-providers-regarding-ebola-virus-outbreak-in-uganda-sudan-ebolavirus

[44] Centers for Disease Control and Prevention. Outbreak of Ebola virus disease (Sudan ebolavirus) in central Uganda. Health Alert Network, 2022. https://emergency.cdc.gov/han/2022/han00477.asp

[45] Adetifa I. Ncdc on alert mode following the outbreak of Ebola virus disease (EVD) detected in Uganda. Nigeria Centre for Disease Control and Prevention, 2022. https://ncdc.gov.ng/news/418/ncdc-on-alert-mode-following-the-outbreak-of-ebola-virus-disease-%28evd%29-detected-in-uganda#

[46] Nigeria centre for disease control and prevention. public health Advisory following Declaration of Ebola virus disease outbreak in Uganda. Recent Publication 2022 https://ncdc.gov.ng/news/423/public-health-advisory-following-declaration-of-ebola-virus-disease-outbreak-in-uganda

[47] Norton A, De La Horra Gozalo A, Feune de Colombi N, et al. The remaining unknowns: a mixed methods study of the current and global health research priorities for COVID-19. BMJ Glob Health 2020;5:e003306. 10.1136/bmjgh-2020-003306 PMC7431769 32727843

[48] Lang T. Ebola: embed research in outbreak response. Nature 2015;524:29–31. 10.1038/524029a 26245565

