# Supplementary material for: Sudan virus disease outbreak in Uganda: urgent research gaps
Source: BMJ Glob Health. 2022 Dec 30;7(12):e010982. doi: 10.1136/bmjgh-2022-010982 (PMC9809242; doi:10.1136/bmjgh-2022-010982)
Supplement: Supplementary data [file bmjgh-2022-010982supp001.pdf]

Appendix 1: SUDV research studies registered as of 15 November 2022

Project title	Start date	End date	Funder	Director	Country	Research theme
**World RePORT database**						
Thermostabilized Subunit Glycoprotein Vaccine Platform: Immune Characterization of an Emulsified	Dec. 2020	Nov. 2022	NIH	Donini, O	USA	Vaccines
A "Trojan Horse" bispecific antibody strategy for broad filovirus therapeutics	Jul. 2017	Jun. 2022	NIH	Chandran, K	USA	Therapeutics
Rationally Designed Pan-Ebolavirus Vaccine	Jun. 2017	May 2022	NIH	Qiu, X; Aman, MJ	Canada; USA	Vaccines
Preclinical Development of a Thermostable Trivalent Filovirus Vaccine	Jun. 2017	May 2022	NIH	Lehrer, AT; Bedwell G	USA; UK	Vaccines
Evolution of anti-filovirus B cell responses and mechanisms of protection	Apr. 2017	Mar. 2022	NIH	Aman, MJ	USA	Vaccines
Engineered, multispecific antibodies as broad anti-filovirus therapeutics	Jan. 2017	May 2021	NIH	Lai, J	USA	Therapeutics
Remodeled glycoprotein for broad protection against ebolaviruses	Jan 2018	Dec. 2020	NIH	Aman, MJ	USA	Vaccines
Broadly Protective Bispecific Antibodies for Treatment of Ebola Virus Disease	May 2016	Jun. 2020	NIH	Aman, MJ	USA	Therapeutics
Consortium for Immunotherapeutics Against Viral Hemorrhagic Fevers	Mar. 2014	Feb. 2019	NIH	Nykiforuk,K; Lobel, B; Lutwama; Khan; Saphire; Happi	Canada, Israel, Argentina, Uganda, Sierra Leone, USA, Nigeria	Therapeutics
Development of CM-SV1, a monoclonal antibody treatment for Sudan Virus	Feb. 2018	Jan. 2020	NIH	Guyre, P	USA	Therapeutics
Transcriptomics of immunity and disease in African Fruit Bats-important zoonotic reservoirs	Jan. 2017	Dec. 2019	NIH	Reeder, DA; Geoffrey, A	USA; Uganda	One Health
SSHAP response to Ebola outbreak in DRC and neighbouring countries	Nov. 2018	Nov. 2019	Wellcome Trust	Bedford, J	UK	Social Sciences
Optimization of Ebola Virus Entry Inhibitors	Jan. 2018	Oct. 2019	NIH	Mccormack, KJ	USA	Therapeutics
Mammalian and Plant-derived Antibody-based Therapies against Sudan Ebolavirus	Jan. 2014	May 2019	NIH	Dye, JM	USA	Therapeutics
Preclinical characterization of a multivalent killed Filovirus/Rabies vaccine	Jan. 2013	Jan. 2019	NIH	Neubert, A; Schnell, MJ	USA; Germany	Vaccines
Structure-guided redesign of monoclonal antibodies targeting conserved filovirus epitopes	Jan. 2016	Jun. 2017	NIH	Aman, MJ	USA	Therapeutics

Recombinant antigen diagnostics for filoviruses	Jan. 2010	May 2017	NIH	Boisen, LM	USA	Diagnostics
Direct sequencing of serum antibodies after infection	Feb. 2017	Oct. 2018	NIH	Guthals, A	USA	Therapeutics
**WHO ICTRP database & EU clinical trial register**						
A Phase I & Ib Study to Determine the Safety and Immunogenicity of a Bivalent ChAdOx1 Vectored Vaccine Against Zaire and Sudan Ebola Virus Species in UK & Tanzanian Healthy Adult Volunteers **Note: This was registered as two separate studies on WHO ICTRP database (Phase I in UK and Phase Ib in Tanzania). Here we have combined them.*	Nov. 2021 (UK); Mar. 2022 (Tanzania)	Recruiting	University of Oxford (UKRI)	Olotu, A	UK; Tanzania	Vaccines
A Phase I Open-Label, Dose-Escalation Clinical Trial to Evaluate the Safety, Tolerability and Immunogenicity of Two Doses of an Ebola Sudan Chimpanzee Adenovirus Vector Vaccine, VRC-EBOADC086-00-VP (cAd3- EBO S), in Healthy Adults	Jul. 2019	Completed	NIAID, Sabin Vaccine Institute (NIH)	Unknown	Uganda	Vaccines
A Phase 2 Randomized, Multi-Center Double-Blind, Placebo-Controlled Study to Evaluate the Safety and Immunogenicity of the V920 (rVSV?G-ZEBOV-GP) Ebola Virus Vaccine Candidate in HIV-Infected Adults and Adolescents	Aug. 2017	Unknown	Dalhousie University (Unknown)	Tremlay, C	Canada + two African sites	Vaccines
**Pan African Clinical Trial Registry**						
No trials registered						
